# (*E*)-2-[(2-Formyl­phen­oxy)meth­yl]-3-(4-iso­propyl­phen­yl)acrylo­nitrile

**DOI:** 10.1107/S1600536813020618

**Published:** 2013-07-31

**Authors:** J. Kanchanadevi, G. Anbalagan, R. Selvakumar, M. Bakthadoss, B. Gunasekaran, V. Manivannan

**Affiliations:** a Department of Physics, Velammal Institute of Technology, Panchetty, Chennai 601 204, India; b Department of Physics, Presidency College (Autonomous), Chennai 600 005, India; c Department of Organic Chemistry, University of Madras, Guindy campus, Chennai 600 025, India; d Department of Physics & Nano Technology, SRM University, SRM Nagar, Kattankulathur, Kancheepuram Dist, Chennai 603 203, Tamil Nadu, India; e Department of Research and Development, PRIST University, Vallam, Thanjavur 613 403, Tamil Nadu, India

## Abstract

In the title compound, C_20_H_19_NO_2_, the dihedral angle between the benzene rings is 77.12 (8)°. The terminal isopropyl group is disordered over two orientations, with site occupancies of 0.720 (14) and 0.280 (14). In the crystal, mol­ecules are linked through a weak C—H⋯O inter­action, forming a zigzag chain along the *c-*axis direction.

## Related literature

For the biological activity of cyano­acrylates, see: Zhang *et al.* (2009 ▶); Obniska *et al.* (2005 ▶). For related structures, see: Ye *et al.* (2009 ▶); Suresh *et al.* (2012 ▶); Govindan *et al.* (2012 ▶).
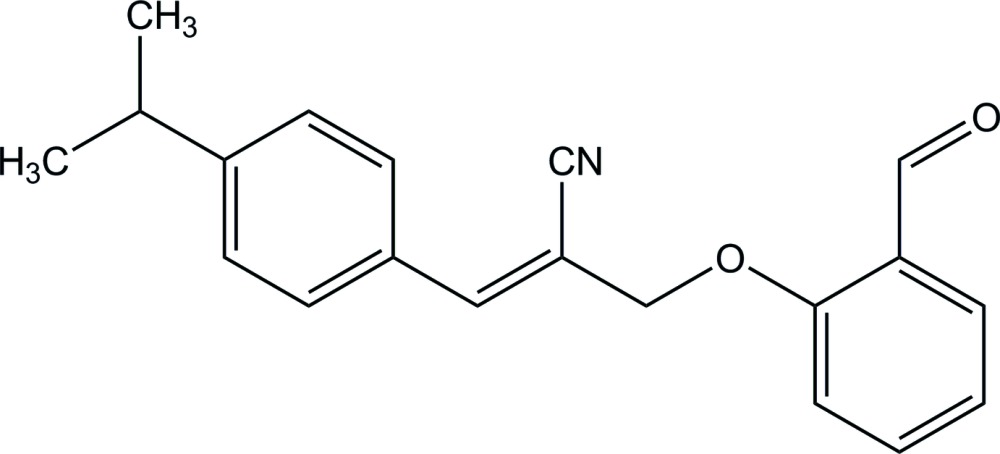


## Experimental

### 

#### Crystal data

C_20_H_19_NO_2_
*M* *_r_* = 305.36
Monoclinic, 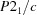

*a* = 13.3276 (9) Å
*b* = 11.6435 (7) Å
*c* = 11.9965 (9) Å
β = 111.800 (3)°
*V* = 1728.5 (2) Å^3^
*Z* = 4
Mo *K*α radiation
μ = 0.08 mm^−1^
*T* = 295 K
0.30 × 0.25 × 0.20 mm

#### Data collection

Bruker APEXII CCD diffractometer
Absorption correction: multi-scan (*SADABS*; Sheldrick, 1996 ▶) *T*_min_ = 0.977, *T*_max_ = 0.985
16125 measured reflections
3532 independent reflections
2004 reflections with *I* > 2σ(*I*)
*R*_int_ = 0.025

#### Refinement

*R*[*F* ^2^ > 2σ(*F* ^2^)] = 0.056
*wR*(*F* ^2^) = 0.179
*S* = 1.09
3532 reflections
231 parameters
12 restraints
H-atom parameters constrained
Δρ_max_ = 0.32 e Å^−3^
Δρ_min_ = −0.18 e Å^−3^

### 

Data collection: *APEX2* (Bruker, 2008 ▶); cell refinement: *SAINT* (Bruker, 2008 ▶); data reduction: *SAINT*; program(s) used to solve structure: *SHELXS97* (Sheldrick, 2008 ▶); program(s) used to refine structure: *SHELXL97* (Sheldrick, 2008 ▶); molecular graphics: *PLATON* (Spek, 2009 ▶); software used to prepare material for publication: *SHELXL97*.

## Supplementary Material

Crystal structure: contains datablock(s) I. DOI: 10.1107/S1600536813020618/is5291sup1.cif

Structure factors: contains datablock(s) I. DOI: 10.1107/S1600536813020618/is5291Isup2.hkl

Supplementary material file. DOI: 10.1107/S1600536813020618/is5291Isup3.cml

Additional supplementary materials:  crystallographic information; 3D view; checkCIF report

## Figures and Tables

**Table 1:** Hydrogen-bond geometry (Å, °)

*D*—H⋯*A*	*D*—H	H⋯*A*	*D*⋯*A*	*D*—H⋯*A*
C3—H3⋯O2^i^	0.93	2.41	3.236 (3)	149

Symmetry code: (i) 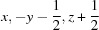.

**Fig. 1.**
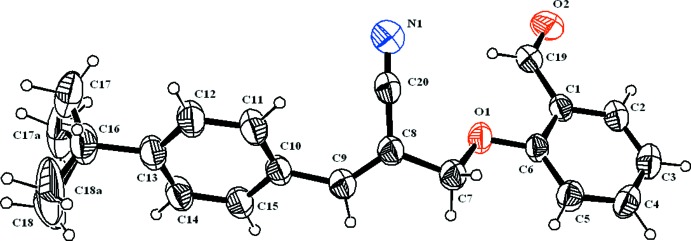
The molecular structure of the title compound, with atom labels and 30% probability displacement ellipsoids for non-H atoms.

**Fig. 2.**
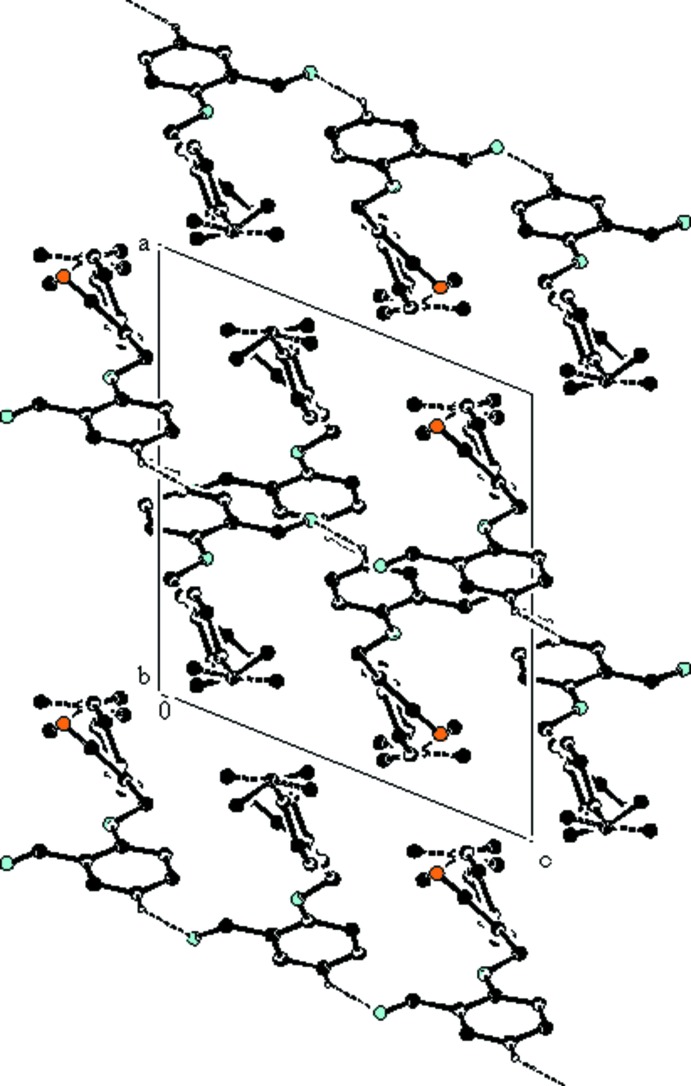
A packing diagram of the title compound, viewed down the b axis. Hydrogen bonds are shown as dashed lines.

## supplementary crystallographic information

### Comment

Cyanoacrylates and its derivatives have been widely used as agrochemicals (Zhang *et al.*, 2009) and an important intermediate in drugs synthesis (Obniska *et al.*, 2005).

The geometric parameters of the title molecule (Fig. 1) agree well with reported similar structure(Ye *et al.*, 2009; Suresh *et al.*, 2012; Govindan *et al.*, 2012). The dihedral angle between the two benzene rings (C1—C6 & C10—C15) is 77.12 (8)°. The terminal methyl groups are disordered over two positions with the site occupancies of C17 = 0.280 (14) and C17A = 0.720 (14), C18 = 0.280 (14) and C18A = 0.720 (14). The crystal packing is through a weak intermolecular C—H···O interaction.

### Experimental

A solution of salicylaldehyde (1 equivalent) and potassium carbonate (1 equivalent) in acetonitrile solvent was stirred for 15 minutes at room temperature. To this solution, (*Z*)-methyl 2-(bromomethyl)-3-(4-isopropylphenyl)acrylate (1 equivalent) was added dropwise. After the completion of the reaction as indicated by TLC, acetonitrile was evaporated. Ethylacetate (15 ml) and water (15 ml) were added to the crude mass and extracted. The organic layer was dried over anhydrous sodium sulfate. Removal of solvent led to the crude product which was purified through pad of silica gel (100–200 mesh) using ethylacetate and hexane (1:9) as solvents. The pure title compound was obtained as colorless solid (94%). Recrystallization was carried out using ethylacetate as solvent.

### Refinement

The site occupancy factors of disordered C atoms in the methyl groups were refined to C17 = 0.280 (14) and C17A = 0.720 (14), C18 = 0.280 (14) and C18A = 0.720 (14). The bond distances C16—C17, C16—C17A, C16—C18 and C16—C18A were restrained to 1.520 (5) Å and the contact distances C17···C18 and C17A···C18A were restrained to 2.48 (1) Å. H atoms were positioned geometrically and refined using riding model with C—H = 0.93 Å and *U*_iso_(H) = 1.2*U*_eq_(C) for aromatic CH, C—H = 0.98 Å and *U*_iso_(H) = 1.2*U*_eq_(C) for methine CH, C—H = 0.97 Å and *U*_iso_(H) = 1.2*U*_eq_(C) for CH_2_ and C—H = 0.96 Å and *U*_iso_(H) = 1.5*U*_eq_(C) for CH_3_. The components of the anisotropic displacement parameters in direction of the bond of C16, C17, C17A and C18A were restrained to be equal within an effective standard deviation of 0.001 using the *DELU* command in *SHELXL* (Sheldrick, 2008).

### Crystal data

**Table:**

C_20_H_19_NO_2_	*F*(000) = 648
*M**_r_* = 305.36	*D*_x_ = 1.173 Mg m^−^^3^
Monoclinic, *P*2_1_/*c*	Mo *K*α radiation, λ = 0.71073 Å
Hall symbol: -P 2ybc	Cell parameters from 3895 reflections
*a* = 13.3276 (9) Å	θ = 2.4–26.4°
*b* = 11.6435 (7) Å	µ = 0.08 mm^−^^1^
*c* = 11.9965 (9) Å	*T* = 295 K
β = 111.800 (3)°	Block, colourless
*V* = 1728.5 (2) Å^3^	0.30 × 0.25 × 0.20 mm
*Z* = 4	

### Data collection

**Table:**

Bruker APEXII CCD diffractometer	3532 independent reflections
Radiation source: fine-focus sealed tube	2004 reflections with *I* > 2σ(*I*)
Graphite monochromator	*R*_int_ = 0.025
Detector resolution: 0 pixels mm^-1^	θ_max_ = 26.4°, θ_min_ = 2.4°
ω and φ scans	*h* = −16→15
Absorption correction: multi-scan (*SADABS*; Sheldrick, 1996)	*k* = −9→14
*T*_min_ = 0.977, *T*_max_ = 0.985	*l* = −15→14
16125 measured reflections	

### Refinement

**Table:**

Refinement on *F*^2^	Primary atom site location: structure-invariant direct methods
Least-squares matrix: full	Secondary atom site location: difference Fourier map
*R*[*F*^2^ > 2σ(*F*^2^)] = 0.056	Hydrogen site location: inferred from neighbouring sites
*wR*(*F*^2^) = 0.179	H-atom parameters constrained
*S* = 1.09	*w* = 1/[σ^2^(*F*_o_^2^) + (0.074*P*)^2^ + 0.3076*P*] where *P* = (*F*_o_^2^ + 2*F*_c_^2^)/3
3532 reflections	(Δ/σ)_max_ = 0.013
231 parameters	Δρ_max_ = 0.32 e Å^−^^3^
12 restraints	Δρ_min_ = −0.18 e Å^−^^3^

### Fractional atomic coordinates and isotropic or equivalent isotropic displacement parameters (Å^2^)

**Table:**

	*x*	*y*	*z*	*U*_iso_*/*U*_eq_	Occ. (<1)
C1	0.55620 (17)	−0.06955 (16)	0.3043 (2)	0.0650 (6)	
C2	0.5111 (2)	−0.16986 (18)	0.3262 (2)	0.0789 (7)	
H2	0.4677	−0.2142	0.2619	0.095*	
C3	0.5290 (2)	−0.2047 (2)	0.4401 (3)	0.0913 (8)	
H3	0.4977	−0.2720	0.4536	0.110*	
C4	0.5937 (2)	−0.1397 (2)	0.5349 (2)	0.0878 (7)	
H4	0.6065	−0.1642	0.6128	0.105*	
C5	0.64009 (19)	−0.0388 (2)	0.5171 (2)	0.0777 (6)	
H5	0.6837	0.0046	0.5822	0.093*	
C6	0.62042 (17)	−0.00323 (17)	0.40049 (19)	0.0635 (5)	
C7	0.7365 (2)	0.16085 (19)	0.4660 (2)	0.0791 (7)	
H7A	0.7974	0.1128	0.5116	0.095*	
H7B	0.7036	0.1903	0.5200	0.095*	
C8	0.77323 (18)	0.25785 (18)	0.4086 (2)	0.0725 (6)	
C9	0.76564 (19)	0.36663 (19)	0.4379 (2)	0.0759 (6)	
H9	0.7331	0.3771	0.4935	0.091*	
C10	0.79995 (19)	0.47258 (18)	0.3971 (2)	0.0716 (6)	
C11	0.8839 (2)	0.4797 (2)	0.3558 (3)	0.0899 (8)	
H11	0.9195	0.4132	0.3482	0.108*	
C12	0.9151 (2)	0.5837 (2)	0.3258 (3)	0.0987 (9)	
H12	0.9723	0.5859	0.2991	0.118*	
C13	0.8652 (2)	0.6843 (2)	0.3339 (2)	0.0886 (8)	
C14	0.7801 (2)	0.6776 (2)	0.3723 (2)	0.0923 (8)	
H14	0.7429	0.7441	0.3760	0.111*	
C15	0.7491 (2)	0.5741 (2)	0.4052 (2)	0.0852 (7)	
H15	0.6929	0.5725	0.4335	0.102*	
C16	0.9055 (3)	0.7981 (2)	0.3044 (3)	0.1238 (10)	
H16A	0.9825	0.7830	0.3239	0.149*	0.280 (14)
H16B	0.9563	0.7792	0.2653	0.149*	0.720 (14)
C17	0.8674 (13)	0.8319 (18)	0.1750 (6)	0.168 (8)	0.280 (14)
H17A	0.9165	0.8870	0.1640	0.252*	0.280 (14)
H17B	0.7967	0.8652	0.1512	0.252*	0.280 (14)
H17C	0.8646	0.7652	0.1269	0.252*	0.280 (14)
C18	0.9075 (17)	0.8975 (11)	0.3860 (14)	0.158 (7)	0.280 (14)
H18A	0.9376	0.8725	0.4681	0.237*	0.280 (14)
H18B	0.8351	0.9247	0.3680	0.237*	0.280 (14)
H18C	0.9509	0.9584	0.3738	0.237*	0.280 (14)
C18A	0.9463 (8)	0.8730 (6)	0.4109 (6)	0.222 (5)	0.720 (14)
H18D	0.9905	0.9324	0.3975	0.333*	0.720 (14)
H18E	0.9886	0.8283	0.4796	0.333*	0.720 (14)
H18F	0.8865	0.9069	0.4250	0.333*	0.720 (14)
C17A	0.8161 (7)	0.8571 (7)	0.2060 (7)	0.230 (6)	0.720 (14)
H17D	0.7543	0.8641	0.2284	0.344*	0.720 (14)
H17E	0.7971	0.8129	0.1335	0.344*	0.720 (14)
H17F	0.8396	0.9321	0.1931	0.344*	0.720 (14)
C19	0.5360 (2)	−0.0363 (2)	0.1806 (2)	0.0807 (7)	
H19	0.5687	0.0304	0.1678	0.097*	
N1	0.8430 (3)	0.1946 (2)	0.2453 (3)	0.1215 (9)	
C20	0.8136 (2)	0.2253 (2)	0.3180 (3)	0.0851 (7)	
O1	0.65982 (13)	0.09621 (12)	0.37184 (13)	0.0776 (5)	
O2	0.47952 (17)	−0.08980 (16)	0.09395 (17)	0.1076 (7)	

### Atomic displacement parameters (Å^2^)

**Table:**

	*U*^11^	*U*^22^	*U*^33^	*U*^12^	*U*^13^	*U*^23^
C1	0.0713 (13)	0.0488 (11)	0.0771 (14)	0.0039 (10)	0.0303 (11)	0.0005 (10)
C2	0.0840 (16)	0.0558 (13)	0.0971 (18)	−0.0030 (11)	0.0340 (13)	0.0002 (12)
C3	0.0973 (19)	0.0639 (15)	0.117 (2)	−0.0050 (14)	0.0445 (17)	0.0178 (15)
C4	0.0975 (18)	0.0803 (17)	0.0898 (18)	0.0110 (15)	0.0396 (15)	0.0307 (14)
C5	0.0841 (16)	0.0692 (15)	0.0744 (15)	0.0010 (12)	0.0231 (12)	0.0090 (11)
C6	0.0716 (13)	0.0481 (11)	0.0716 (14)	0.0064 (10)	0.0274 (11)	0.0078 (10)
C7	0.0967 (17)	0.0662 (14)	0.0671 (14)	−0.0158 (12)	0.0218 (12)	−0.0022 (11)
C8	0.0844 (15)	0.0597 (13)	0.0679 (13)	−0.0100 (11)	0.0219 (12)	−0.0026 (10)
C9	0.0844 (15)	0.0643 (14)	0.0776 (15)	−0.0101 (12)	0.0286 (12)	−0.0049 (11)
C10	0.0784 (15)	0.0560 (13)	0.0769 (14)	−0.0072 (11)	0.0250 (12)	−0.0054 (10)
C11	0.0912 (18)	0.0581 (14)	0.128 (2)	−0.0027 (12)	0.0492 (17)	−0.0043 (13)
C12	0.105 (2)	0.0661 (16)	0.146 (3)	−0.0101 (14)	0.071 (2)	−0.0057 (15)
C13	0.0998 (19)	0.0587 (15)	0.115 (2)	−0.0108 (13)	0.0488 (16)	−0.0037 (13)
C14	0.1020 (19)	0.0552 (14)	0.124 (2)	0.0000 (13)	0.0466 (17)	−0.0065 (13)
C15	0.0939 (17)	0.0669 (15)	0.1036 (19)	−0.0067 (13)	0.0468 (15)	−0.0088 (13)
C16	0.161 (3)	0.0624 (16)	0.180 (3)	−0.0003 (17)	0.101 (2)	0.0119 (16)
C17	0.130 (12)	0.218 (19)	0.161 (5)	−0.096 (12)	0.060 (8)	0.002 (8)
C18	0.25 (2)	0.081 (7)	0.209 (13)	0.058 (11)	0.160 (16)	0.023 (7)
C18A	0.260 (9)	0.100 (4)	0.178 (4)	−0.115 (6)	−0.066 (6)	0.022 (3)
C17A	0.268 (9)	0.191 (7)	0.136 (5)	−0.145 (6)	−0.034 (5)	0.096 (5)
C19	0.0988 (18)	0.0642 (14)	0.0810 (17)	−0.0059 (13)	0.0354 (14)	−0.0101 (12)
N1	0.174 (3)	0.0914 (17)	0.124 (2)	−0.0213 (17)	0.084 (2)	−0.0193 (15)
C20	0.111 (2)	0.0588 (14)	0.0874 (17)	−0.0155 (13)	0.0386 (16)	−0.0045 (13)
O1	0.0981 (11)	0.0565 (9)	0.0699 (9)	−0.0167 (8)	0.0214 (8)	0.0037 (7)
O2	0.1335 (17)	0.1036 (14)	0.0868 (13)	−0.0221 (12)	0.0422 (12)	−0.0279 (10)

### Geometric parameters (Å, º)

**Table:**

C1—C2	1.383 (3)	C13—C14	1.376 (4)
C1—C6	1.388 (3)	C13—C16	1.520 (3)
C1—C19	1.459 (3)	C14—C15	1.379 (3)
C2—C3	1.359 (3)	C14—H14	0.9300
C2—H2	0.9300	C15—H15	0.9300
C3—C4	1.371 (4)	C16—C18A	1.474 (4)
C3—H3	0.9300	C16—C17	1.495 (5)
C4—C5	1.381 (3)	C16—C17A	1.497 (4)
C4—H4	0.9300	C16—C18	1.510 (5)
C5—C6	1.388 (3)	C16—H16A	0.9800
C5—H5	0.9300	C16—H16B	0.9800
C6—O1	1.367 (2)	C17—H17A	0.9600
C7—O1	1.425 (3)	C17—H17B	0.9600
C7—C8	1.496 (3)	C17—H17C	0.9600
C7—H7A	0.9700	C18—H18A	0.9600
C7—H7B	0.9700	C18—H18B	0.9600
C8—C9	1.328 (3)	C18—H18C	0.9600
C8—C20	1.432 (4)	C18A—H18D	0.9600
C9—C10	1.462 (3)	C18A—H18E	0.9600
C9—H9	0.9300	C18A—H18F	0.9600
C10—C15	1.384 (3)	C17A—H17D	0.9600
C10—C11	1.384 (3)	C17A—H17E	0.9600
C11—C12	1.371 (3)	C17A—H17F	0.9600
C11—H11	0.9300	C19—O2	1.206 (3)
C12—C13	1.368 (3)	C19—H19	0.9300
C12—H12	0.9300	N1—C20	1.138 (3)
			
C2—C1—C6	119.3 (2)	C13—C14—H14	119.4
C2—C1—C19	119.3 (2)	C15—C14—H14	119.4
C6—C1—C19	121.45 (19)	C14—C15—C10	121.3 (2)
C3—C2—C1	121.1 (2)	C14—C15—H15	119.3
C3—C2—H2	119.5	C10—C15—H15	119.3
C1—C2—H2	119.5	C18A—C16—C17A	109.9 (4)
C2—C3—C4	119.4 (2)	C17—C16—C18	112.9 (7)
C2—C3—H3	120.3	C18A—C16—C13	111.2 (4)
C4—C3—H3	120.3	C17—C16—C13	117.3 (8)
C3—C4—C5	121.4 (2)	C17A—C16—C13	109.7 (3)
C3—C4—H4	119.3	C18—C16—C13	116.1 (7)
C5—C4—H4	119.3	C18A—C16—H16A	83.2
C4—C5—C6	118.8 (2)	C17—C16—H16A	102.6
C4—C5—H5	120.6	C17A—C16—H16A	136.7
C6—C5—H5	120.6	C18—C16—H16A	102.6
O1—C6—C5	124.0 (2)	C13—C16—H16A	102.6
O1—C6—C1	115.98 (18)	C18A—C16—H16B	116.7
C5—C6—C1	120.0 (2)	C17A—C16—H16B	102.4
O1—C7—C8	107.17 (18)	C13—C16—H16B	106.4
O1—C7—H7A	110.3	C16—C17—H17A	109.5
C8—C7—H7A	110.3	C16—C17—H17B	109.5
O1—C7—H7B	110.3	C16—C17—H17C	109.5
C8—C7—H7B	110.3	C16—C18—H18A	109.5
H7A—C7—H7B	108.5	C16—C18—H18B	109.5
C9—C8—C20	122.7 (2)	C16—C18—H18C	109.5
C9—C8—C7	121.9 (2)	C16—C18A—H18D	109.5
C20—C8—C7	115.40 (19)	C16—C18A—H18E	109.5
C8—C9—C10	130.7 (2)	H18D—C18A—H18E	109.5
C8—C9—H9	114.6	C16—C18A—H18F	109.5
C10—C9—H9	114.6	H18D—C18A—H18F	109.5
C15—C10—C11	117.1 (2)	H18E—C18A—H18F	109.5
C15—C10—C9	118.4 (2)	C16—C17A—H17D	109.5
C11—C10—C9	124.4 (2)	C16—C17A—H17E	109.5
C12—C11—C10	120.7 (2)	H17D—C17A—H17E	109.5
C12—C11—H11	119.6	C16—C17A—H17F	109.5
C10—C11—H11	119.6	H17D—C17A—H17F	109.5
C13—C12—C11	122.4 (2)	H17E—C17A—H17F	109.5
C13—C12—H12	118.8	O2—C19—C1	124.0 (2)
C11—C12—H12	118.8	O2—C19—H19	118.0
C12—C13—C14	117.2 (2)	C1—C19—H19	118.0
C12—C13—C16	120.5 (2)	N1—C20—C8	176.6 (3)
C14—C13—C16	122.3 (2)	C6—O1—C7	118.54 (16)
C13—C14—C15	121.2 (2)		
			
C6—C1—C2—C3	−0.3 (3)	C11—C12—C13—C14	−0.8 (5)
C19—C1—C2—C3	179.2 (2)	C11—C12—C13—C16	177.6 (3)
C1—C2—C3—C4	−0.5 (4)	C12—C13—C14—C15	2.2 (4)
C2—C3—C4—C5	0.8 (4)	C16—C13—C14—C15	−176.1 (3)
C3—C4—C5—C6	−0.1 (4)	C13—C14—C15—C10	−2.2 (4)
C4—C5—C6—O1	178.4 (2)	C11—C10—C15—C14	0.7 (4)
C4—C5—C6—C1	−0.8 (3)	C9—C10—C15—C14	177.7 (2)
C2—C1—C6—O1	−178.29 (18)	C12—C13—C16—C18A	−115.9 (6)
C19—C1—C6—O1	2.2 (3)	C14—C13—C16—C18A	62.4 (7)
C2—C1—C6—C5	1.0 (3)	C12—C13—C16—C17	83.0 (9)
C19—C1—C6—C5	−178.5 (2)	C14—C13—C16—C17	−98.7 (9)
O1—C7—C8—C9	124.1 (2)	C12—C13—C16—C17A	122.2 (6)
O1—C7—C8—C20	−54.1 (3)	C14—C13—C16—C17A	−59.5 (7)
C20—C8—C9—C10	−4.7 (4)	C12—C13—C16—C18	−139.3 (10)
C7—C8—C9—C10	177.3 (2)	C14—C13—C16—C18	39.0 (10)
C8—C9—C10—C15	156.3 (3)	C2—C1—C19—O2	1.7 (4)
C8—C9—C10—C11	−26.9 (4)	C6—C1—C19—O2	−178.8 (2)
C15—C10—C11—C12	0.7 (4)	C5—C6—O1—C7	8.2 (3)
C9—C10—C11—C12	−176.1 (2)	C1—C6—O1—C7	−172.53 (19)
C10—C11—C12—C13	−0.7 (5)	C8—C7—O1—C6	174.72 (18)

### Hydrogen-bond geometry (Å, º)

**Table:**

*D*—H···*A*	*D*—H	H···*A*	*D*···*A*	*D*—H···*A*
C3—H3···O2^i^	0.93	2.41	3.236 (3)	149

Symmetry code: (i) *x*, −*y*−1/2, *z*+1/2.
